# Effects of Transgenic Insect-Resistant Maize HGK60 on Rhizosphere Soil Bacterial Communities

**DOI:** 10.3390/microorganisms13081892

**Published:** 2025-08-14

**Authors:** Yanjun Chen, Junyi Yang, Libo Pan, Meng Liu, Qiuming Wang, Nengwen Xiao, Xiao Guan

**Affiliations:** 1 State Environmental Protection Key Laboratory of Regional Eco-Process and Function Assessment, Chinese Research Academy of Environmental Sciences, Beijing 100012, China; 2 School of Ecology & Environment, Renmin University of China, Beijing 100782, China; yangjunyi20@mails.ucas.ac.cn

**Keywords:** transgenic, bacteria, community structure, diversity, reproductive period

## Abstract

While genetically modified crops bring significant economic benefits, the environmental safety issues they may pose have also received increasing attention. To study the impact of planting genetically modified insect-resistant crops on soil ecosystems, this research employed methods such as 16S rDNA amplicon full-length sequencing, using transgenic *Cry1Ah* insect-resistant corn HGK60 and its conventional counterpart Zheng 58 as subjects for a three-year continuous survey to analyze the effects of planting transgenic *Cry1Ah* insect-resistant corn HGK60 on the rhizosphere bacterial community. The following results were obtained. (1) A total of 216 corn rhizosphere soil samples were annotated to 51 phyla, 119 orders, 221 families, and 549 genera. (2) Overall, there was no significant difference in the composition of the rhizosphere bacterial community between HGK60 and Zheng 58 at the phylum, class, order, or family levels (*p* > 0.05), and the planting of HGK60 did not significantly affect the relative abundance of rhizosphere probiotics (*p* > 0.05). Some differences appeared only briefly and were not reproducible. (3) Alpha and beta diversity analyses showed that overall, the planting of HGK60 had no significant impact on the structure of the rhizosphere bacterial community (*p* > 0.05). (4) Significant changes in the rhizosphere bacterial community were observed across different growth stages of corn. It can be concluded that the planting of HGK60 has no significant impact on the rhizosphere bacteria. This study provides valuable data support for the environmental safety assessment of genetically modified crops.

## 1. Introduction

Food security is one of the most critical issues of this century [1]. As the fastest-growing biotechnology in modern times, genetically modified biotechnology plays a crucial role in addressing food problems. Genetically modified crops, which introduce beneficial genes into conventional crops, can provide sustainable agricultural and economic benefits for human society [2]. Since the first commercial planting of genetically modified crops in 1996, various types of genetically modified crops have been developed, including insect-resistant, herbicide-tolerant, virus-resistant, and dual-resistant crops [3,4]. The latest report shows that the planting area of genetically modified crops has increased from 1.7 million hectares in 1996 to 202.2 million hm^2^ in 2022, a growth of about 112 times, significantly enhancing global crop productivity [5].

Genetically modified crops, while bringing significant economic benefits, have increasingly attracted attention for their potential environmental safety issues. Whether their cultivation affects soil microorganisms has become one of the hot topics in recent studies. Soil microbial communities are a major component of the soil ecosystem [6], such as plant roots harboring a large number of microorganisms, primarily dominated by bacterial communities in their surrounding environment [7]. These rhizosphere bacteria, from plant pathogens to beneficial bacteria, significantly impact host plants, regulating nutrient uptake and host immunity [8,9]. Based on their different effects on plants, rhizosphere bacteria can be categorized into three types: beneficial, harmful, and neutral. Among these, there is a group of bacteria that have special interactions with plant roots and can promote plant growth; these are commonly known as plant rhizosphere probiotics (plant growth-promoting rhizobacteria, PGPR). PGPR have strong colonization capabilities in plant roots [10], inhibiting pathogens in the root zone, and can be used to control fungal, bacterial, and viral diseases [11]. They also promote plant growth, significantly increase seed germination rates, enhance root growth, and increase plant weight and yield [12,13]. PGPR mainly include *Bacillus* spp., *Pseudomonas* spp., *Enterobacter* spp., *Klebsiella* spp., *Azotobacter* spp., and others [12,14,15,16,17].

Transgenic crops, from planting to the end of their entire growth period, see the proteins expressed by exogenous genes in the plant body enter the soil ecosystem through pathways such as root secretion, making the soil ecosystem a significant site for the release of exogenous gene expression products [18]. To date, some studies have examined the impact of transgenic crops on rhizosphere microbial communities, but most have not found significant effects [19,20,21,22]. However, some studies show that compared to non-transgenic crops, there are notable changes in the rhizosphere microbial communities of transgenic crops [23,24]. An increasing number of scientists believe that scientific assessments of whether transgenic crops significantly affect soil microorganisms should follow the “case-by-case principle” [25], systematically analyzing changes in soil microbial diversity at different stages for specific varieties to determine their impact on soil microbial diversity.

*Cry1Ah* is an insecticidal gene from the Bt subspecies, with independent intellectual property rights in China. The Cry1Ah protein exhibits significant toxicity to Lepidoptera insects, showing markedly higher insecticidal efficiency compared to *Cry1Ab* and *Cry1Ac*, but relatively lower toxicity to economic insects such as silkworms [26]. Using the *Cry1Ah* gene, the insect-resistant corn HGK60 was obtained, which has clear molecular characteristics, single-point insertion, and genetic stability. Years of multi-site field trials have demonstrated that its resistance to field pests like corn borers and cotton bollworms is significantly better than that of conventional corn varieties. This study focuses on the insect-resistant corn HGK60 with the *Cry1Ah* gene and its control variety Zheng 58, examining the impact of planting *Cry1Ah* insect-resistant corn HGK60 on rhizosphere soil bacteria. The aim is to provide more detailed data to support the environmental safety assessment of *Cry1Ah* insect-resistant corn HGK60 and contribute to the healthy development of biosafety management.

## 2. Materials and Methods

### 2.1. Overview of the Research Area

The experimental site is located in the Langfang International Agricultural High-Tech Industrial Park (116°36′34″ E, 39°36′10″ N) in Hebei Province, characterized by a warm temperate continental monsoon climate with distinct monsoon features. The average annual temperature is approximately 11.9 °C, and the average annual precipitation is 554.9 mm, with most rainfall concentrated in summer. The average annual sunshine duration is about 2660 h, and the soil types are mainly sandy loam and clay loam.

### 2.2. Test Materials and Experimental Design

The *Cry1Ah*-transferred insect-resistant corn HGK60 (referred to as “HGK60”) and its control conventional corn Zheng 58 (referred to as “Zheng 58”) were provided by the Institute of Biotechnology, Chinese Academy of Agricultural Sciences (Beijing, China). The study employed a randomized block design. Both treatments, HGK60 and Zheng 58, were set up with six replicates (10 m × 10 m) planted in three consecutive seasons: early May 2019, 2020, and 2021. A 1-m isolation zone was established between different materials, and corn was sown using a one-hole-one-seed or one-hole-two-seeds method, maintaining a row spacing of 60 cm and a plant spacing of 25 cm. Field management practices, including irrigation, were consistent with local conventional corn cultivation methods. No chemical fertilizers or insecticides were applied during the growth period of the corn. Corn planting maintained a scientifically reasonable distance from other crops, ensuring that there were no wild relatives within the isolation zone.

### 2.3. Sample Collection

Samples of the rhizosphere soil were collected over six growth stages: pre-sowing, seedling stage, trumpet mouth stage, tasseling stage, full maturity stage, and post-harvest. Avoiding potential influencing factors such as roads, the sampling method followed the five-point method. After removing surface weeds, the corn plants were completely extracted, and their rhizomes were collected using the “shake root method” [27]. The samples were then stored at −80 °C and 4 °C for high-throughput sequencing.

### 2.4. Full-Length Sequencing of 16S rDNA Amplification

(1) DNA extraction: The FastDNA ^®^ SPIN Kit for soil (MP Biomedicals, Santa Ana, CA, USA) was used to extract the total DNA of maize rhizosphere soil microorganisms.

(2) PCR amplification. Region: V1 to V9 full length; template: diluted genomic DNA; Primers: 8F (5′-AGAGTTTGATCCTGGCTCAG-3′), 1509R (5′-GNTACCTTGTTACGACTT-3′); enzyme and buffer from TransStart^®^ FastPfu DNA Polymerase (TransGen Biotech, Beijing, China); PCR reaction system (50 μL): 1 μL Trans Fastpfu,10 μL 5 × buffer, 5 μL 5 × StimuLate, 5 μL dNTPs (2.5 × 10^−3^ mol/L each), 2 μL primer mix (1 μmol/L), 1 μL gDNA, 26 μL NFW. PCR reaction conditions: pre-denaturation at 98 °C (2 min), denaturation at 95 °C (30 s), annealing at 60 °C (45 s), extension at 72 °C (90 s), followed by 35 cycles, then 10 min at 72 °C for final extension. After mixing samples, electrophoresis was performed using agarose gel (1 × TAE concentration 2%) to purify the PCR product, and the target band was recovered by gel cutting. The reagent kit used was the QIAquick@Gel Extraction Kit gel recovery kit (Qiagen, Düsseldorf, Germany).

(3) High-throughput sequencing: The library was constructed using the SMRTbellTM Template Prep Kit (Pacific Biosciences, Menlo Park, CA, USA). The fragment size was quantified by Qubit and detected by FEMTO Pulse. After qualification, 16S full-length rDNA sequencing was performed. This research was assisted by Beijing Novogene Bioinformatics Technology Co., Ltd. (Beijing, China).

### 2.5. Statistical Analysis of Diversity and Structure of Bacterial Communities

The alpha diversity index can effectively reflect the number, abundance, and distribution of species in a community. Common indices of alpha diversity include the Shannon index, Simpson’s index, PD index, coverage index, etc.

### 2.6. Data Processing and Analysis

(1) Sequencing data processing: After the data were downloaded, CCS (SMRT Link v7.0) was used to correct the sequences, perform SSR filtering, and remove primers to obtain the final valid data. (2) OTU Clustering and Species Annotation: The obtained valid data were clustered using Uparse software (Uparse v7.0.1001) with 97% identity to generate OTUs (operational taxonomic units). Species annotations for each OTU were performed using the Mothur method at various taxonomic levels, and normalization was applied to each sample based on minimum data volume. (3) Sample complexity analysis (alpha diversity): Dilution curves were plotted using R software (Version 2.15.3); alpha diversity indices were calculated using Qiime software (Version 1.9.1). (4) Comparative analysis of multiple samples (beta diversity): Principal coordinate analysis (PCoA), similarity analysis (analysis of similarities, Anosim), and non-weighted group average analysis based on the Bray–Curtis distance (unweighted pair group method with arithmetic mean, UPGMA) were all carried out using R software (Version 2.15.3).

## 3. Results

**Figure 1:**
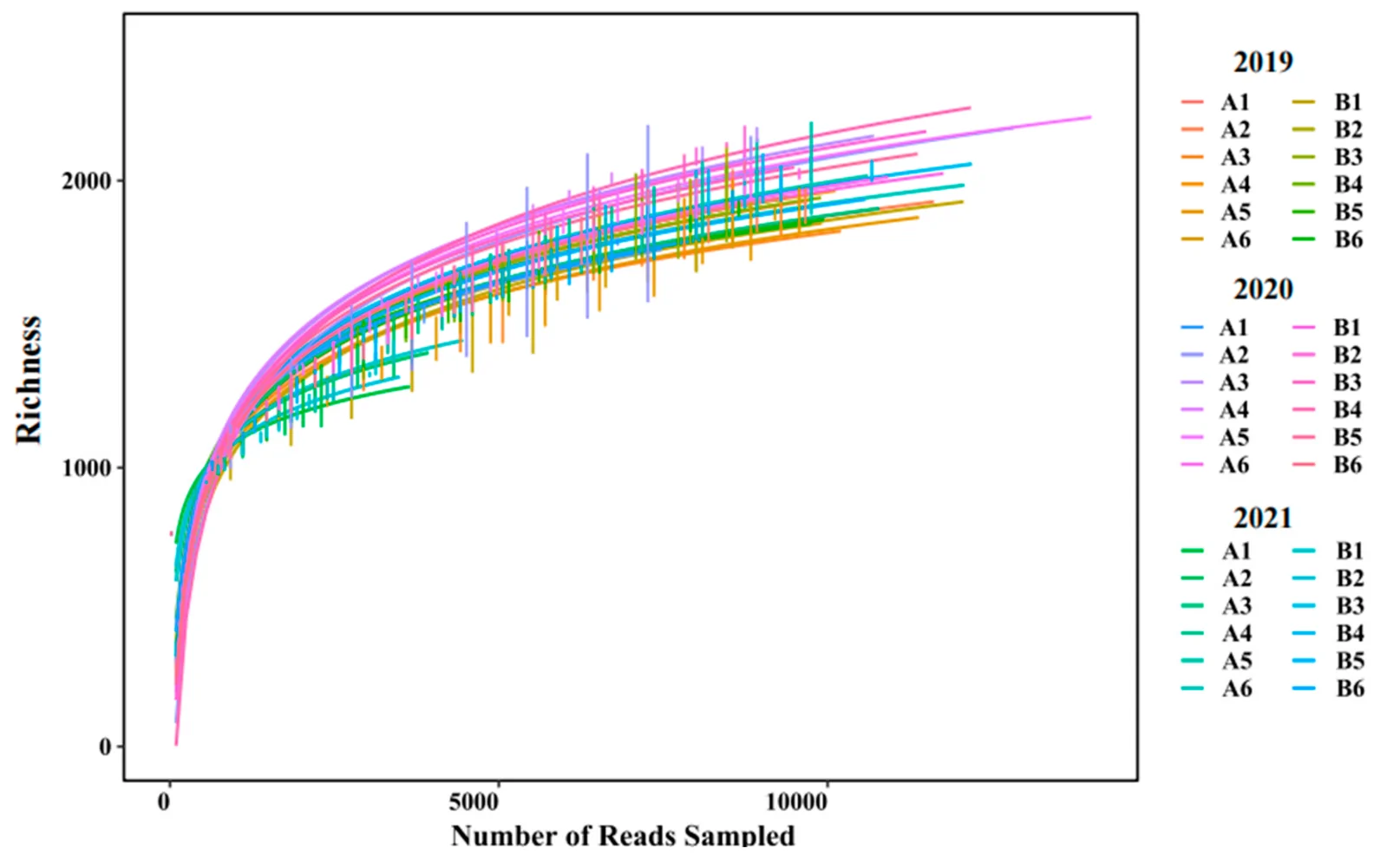
Rarefaction curve.

**Figure 2:**
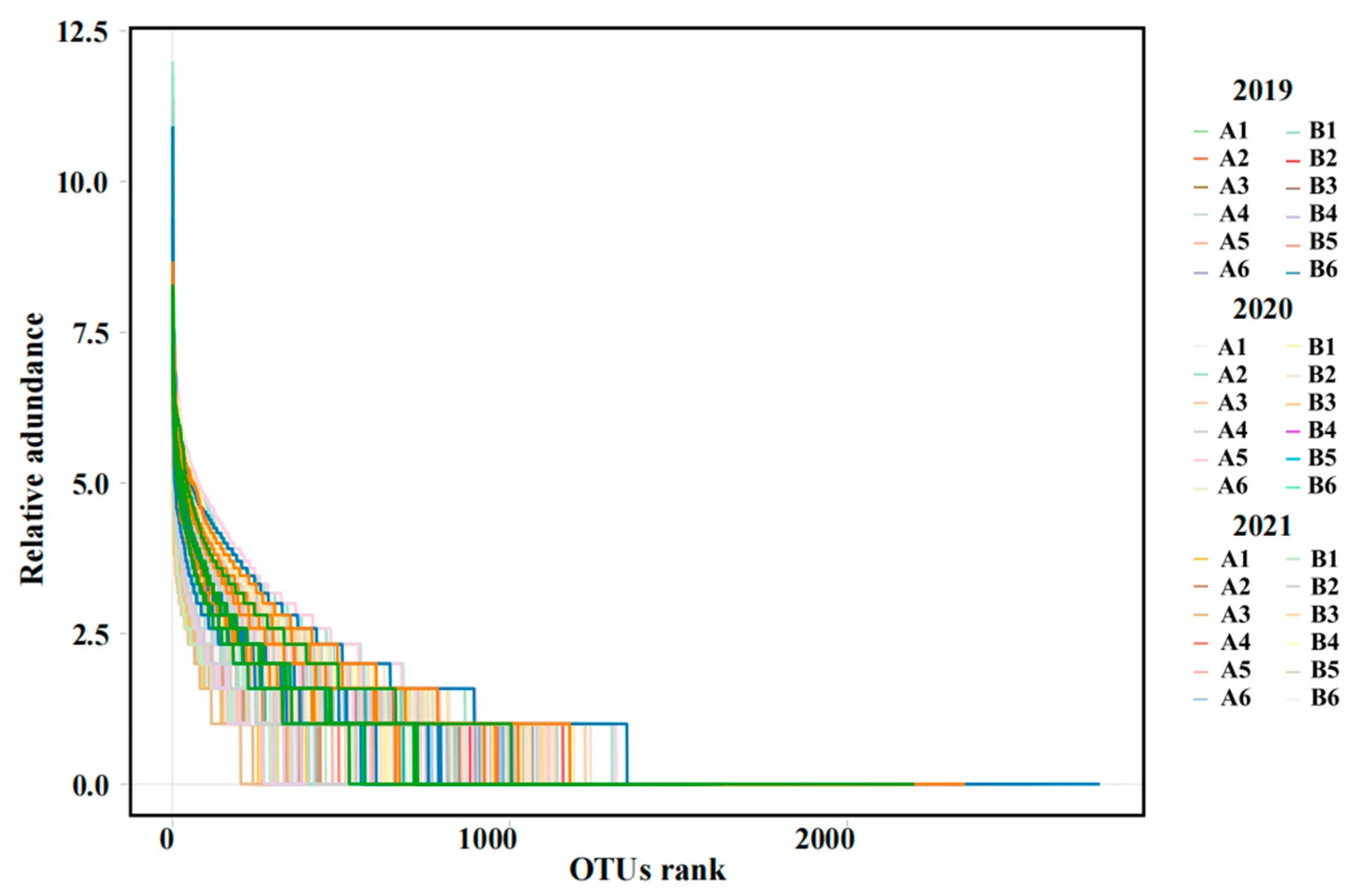
Rank abundance.

**Figure 3:**
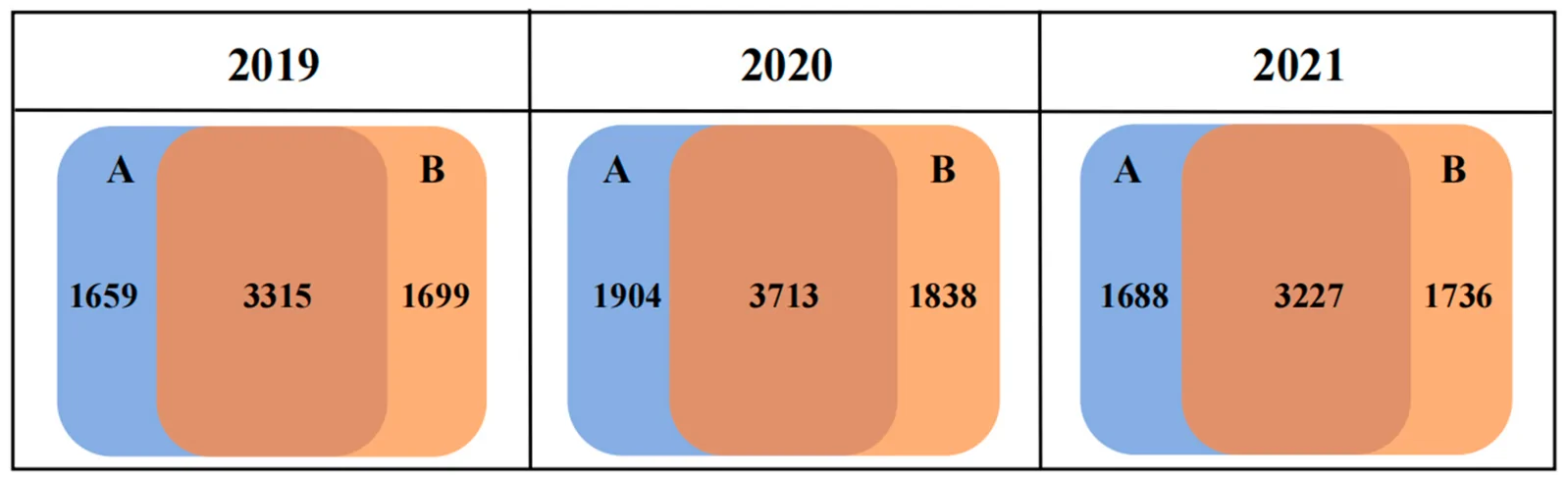
Venn diagrams. A: HGK60, B: Zheng 58. The same below.

High-throughput sequencing was performed on 216 soil samples, clustering them into 10,673 OTUs with 97% identity. The corn rhizosphere soil samples were annotated to 51 phyla, 60 classes, 119 orders, 221 families, and 549 genera. Both the dilution curve (Figure 1) and the abundance rank curve (Figure 2) showed gradual flattening as the sequencing volume increased, indicating uniform species distribution and progressively reasonable data volumes, suitable for subsequent scientific analysis. In 2019, the number of OTUs shared between HGK60 and Zheng 58 was 3315; HGK60 had 1659 OTUs, and Zheng 58 had 1699 OTUs. In 2020, the number of OTUs shared between HGK60 and Zheng 58 was 3713; HGK60 had 1904 OTUs, and Zheng 58 had 1938 OTUs. In 2021, the number of OTUs shared between HGK60 and Zheng 58 was 3227; HGK60 had 1688 OTUs, and Zheng 58 had 1736 OTUs (Figure 3).

### 3.1. Relative Abundance of Rhizosphere Soil Bacterial Species

**Figure 4:**
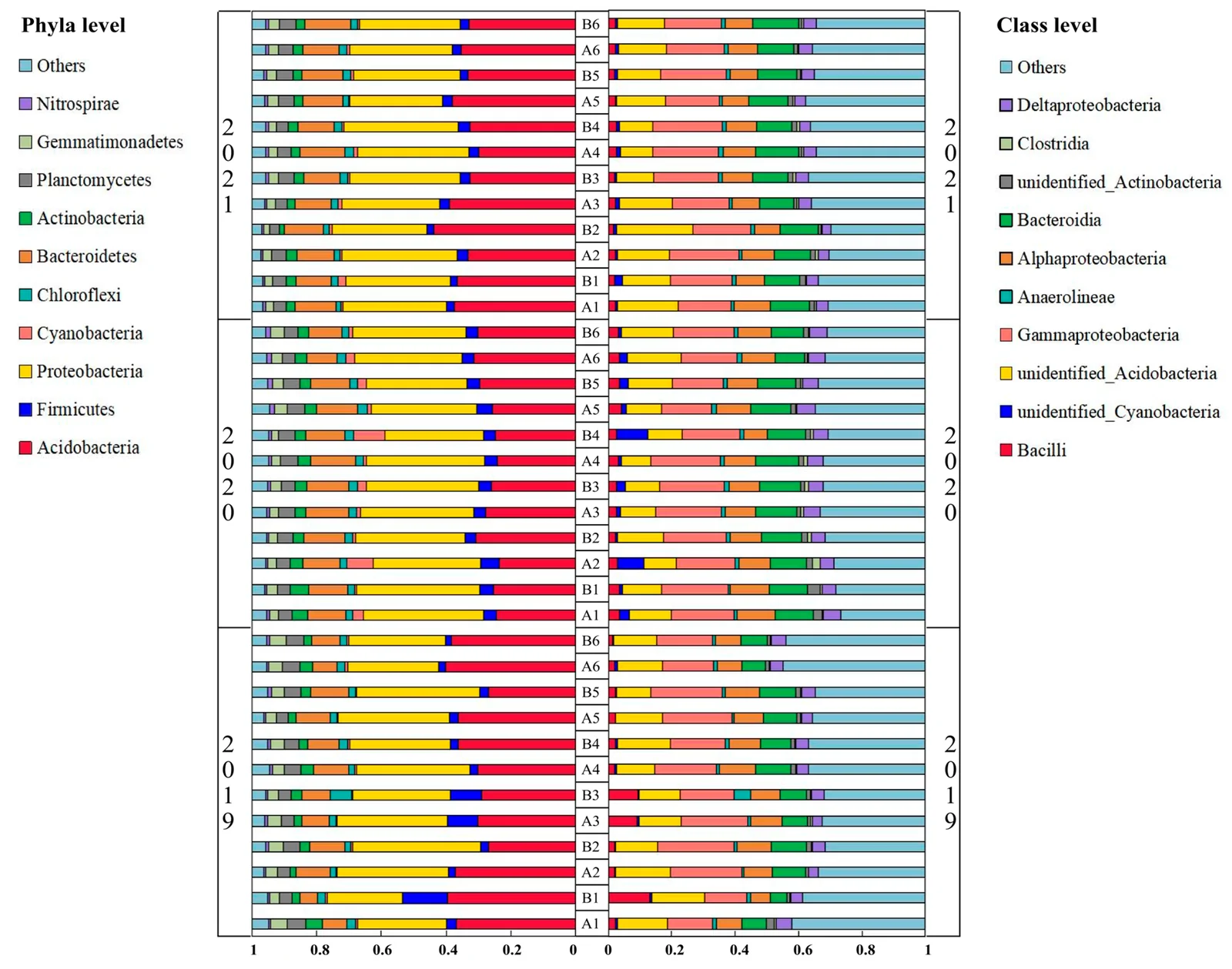
The relative abundance of the top 10 soil bacteria. 1: Pre-planting stage, 2: Seeding stage, 3: Bell stage, 4: Heading stage, 5: Fully ripe stage, 6: Post-harvest stage. The same below.

At the phylum level, the relative abundance of rhizosphere soil bacteria in each year and reproductive stage was analyzed. Proteobacteria and Acidobacteria accounted for more than 15% in all samples during each period, making them dominant bacterial groups. Next were Firmicutes, Cyanobacteria, Chloroflexi, Bacteroidetes, Actinobacteria, Gemmatimonadetes, Planctomycetes, and Nitrospirae (Figure 4). At the class level, Gammaproteobacteria, Bacilli, unidentified Cyanobacteria, unidentified Acidobacteria, Anaerolineae, Bacteroidia, Acidobacteria, Alphaproteobacteria, unidentified Actinobacteria, and Clostridia were the top 10 species in relative abundance (Figure 4). Overall, the relative abundance of rhizosphere soil bacteria at the phylum and class levels between HGK60 and Zheng 58 during the same growth period of corn in the same season was not significantly different (*p* > 0.05). Differences at either the phylum or class levels only briefly appeared during a specific growth period and did not recur across multiple growth periods. For example, cyanobacteria showed significant differences between HGK60 and Zheng 58 during the seedling stage and earing stage in 2020 (*p* < 0.05), but these differences did not appear during the various growth periods in 2019 and 2021 and were not significant after the harvest of three seasons of corn (*p* > 0.05).

**Figure 5:**
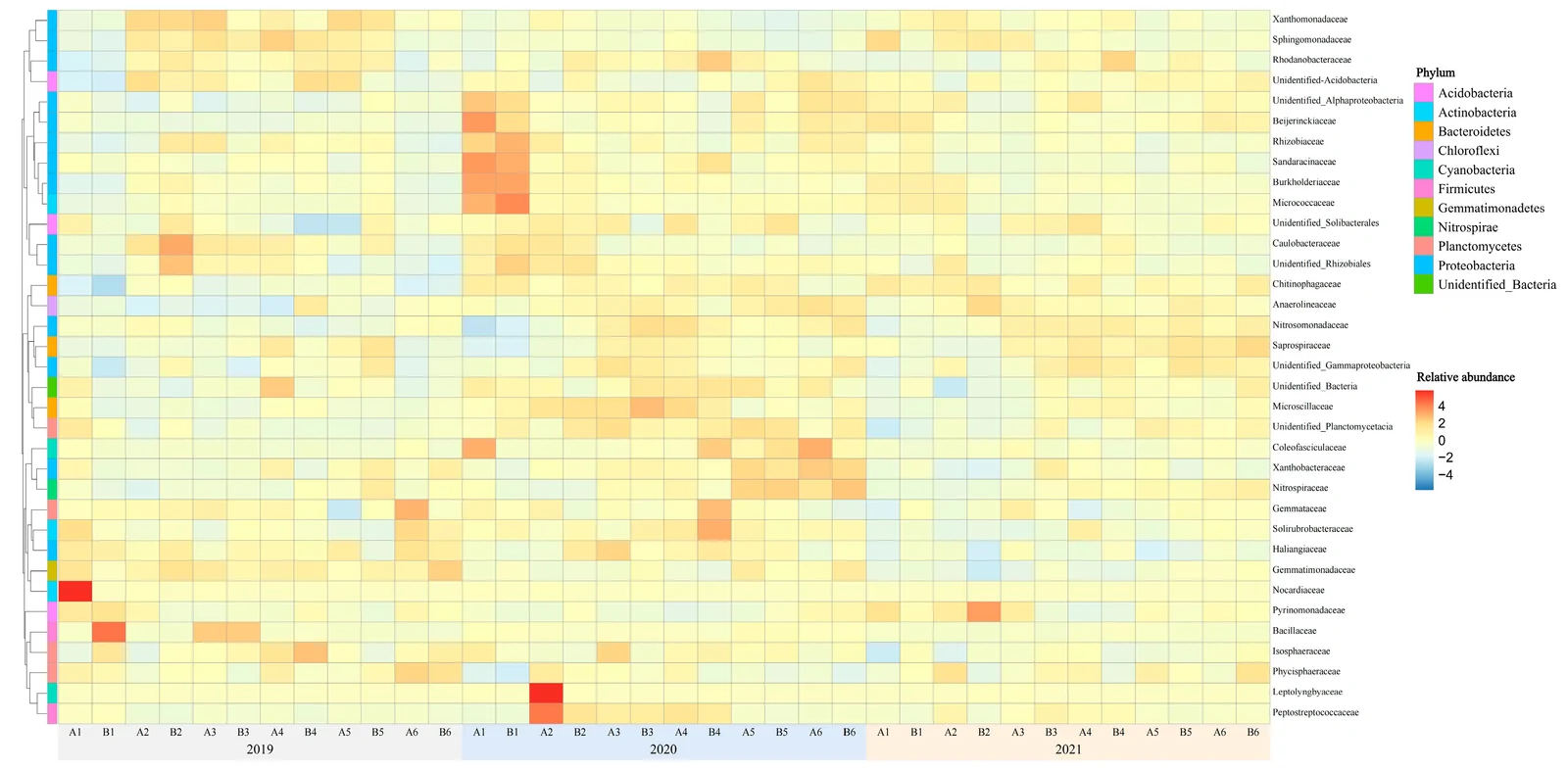
Cluster heatmap of the relative abundance of bacteria at the family level.

**Figure 6:**
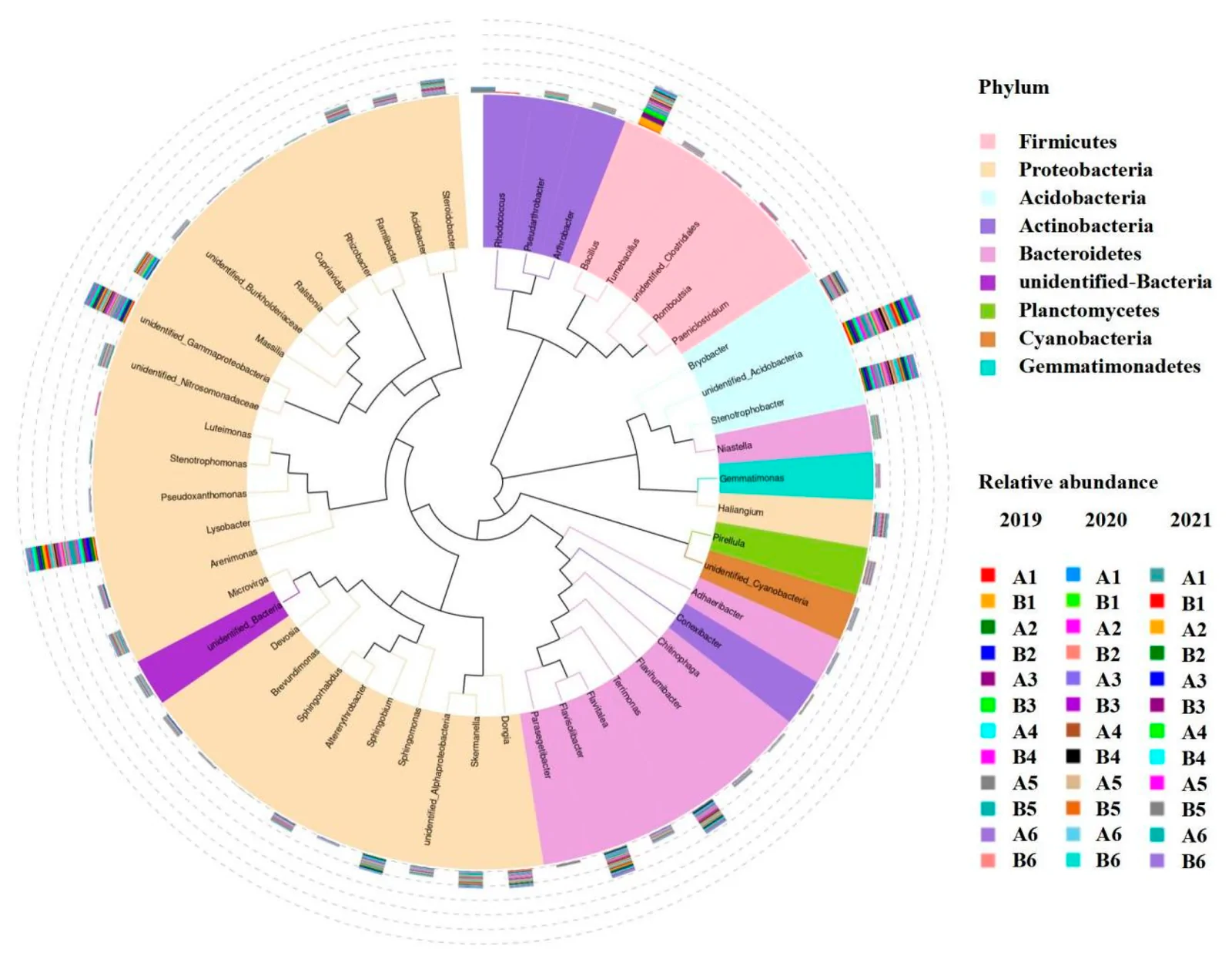
The phylogenetic tree at the genus level.

A clustering heatmap of relative abundance was created for the top 35 families (Figure 5), and a species phylogenetic tree was drawn for the top 100 genera (Figure 6). The species abundance of HGK60 and Zheng 58 at the family level and genus level during the same growth period was compared. Overall, there were no significant differences between HGK60 and Zheng 58. The brief differences that appeared did not recur during the survey, such as in the subfamily Microcystis (Leptolyngbyaceae) and the family Digestion Actinomycetes (Peptostreptococcaceae), which showed significant differences only between HGK60 and Zheng 58 during the seedling stage in 2020; the genus Sphingorhabdus only showed a significant difference in 2019 during the trumpet mouth stage, with HGK60 being significantly lower than Zheng 58; the genera Coniobacter (Conexibacter), Parasegetibacter, Luteimonas, and Pseudococcus (Paeniclostridium) only showed significant differences between HGK60 and Zheng 58 during a certain growth period in 2020; and the genus Rhizoctonia (Ralstonia) only showed a significant difference in 2021 during the full maturity stage, with HGK60 being significantly higher than Zheng 58. Although these families or genera experienced significant increases or decreases in relative abundance at some stages, this change did not persist across all growth periods over three consecutive years. It is believed that these brief differences are more likely due to uncontrollable factors in the field rather than the result of planting HGK60.

**Figure 7:**
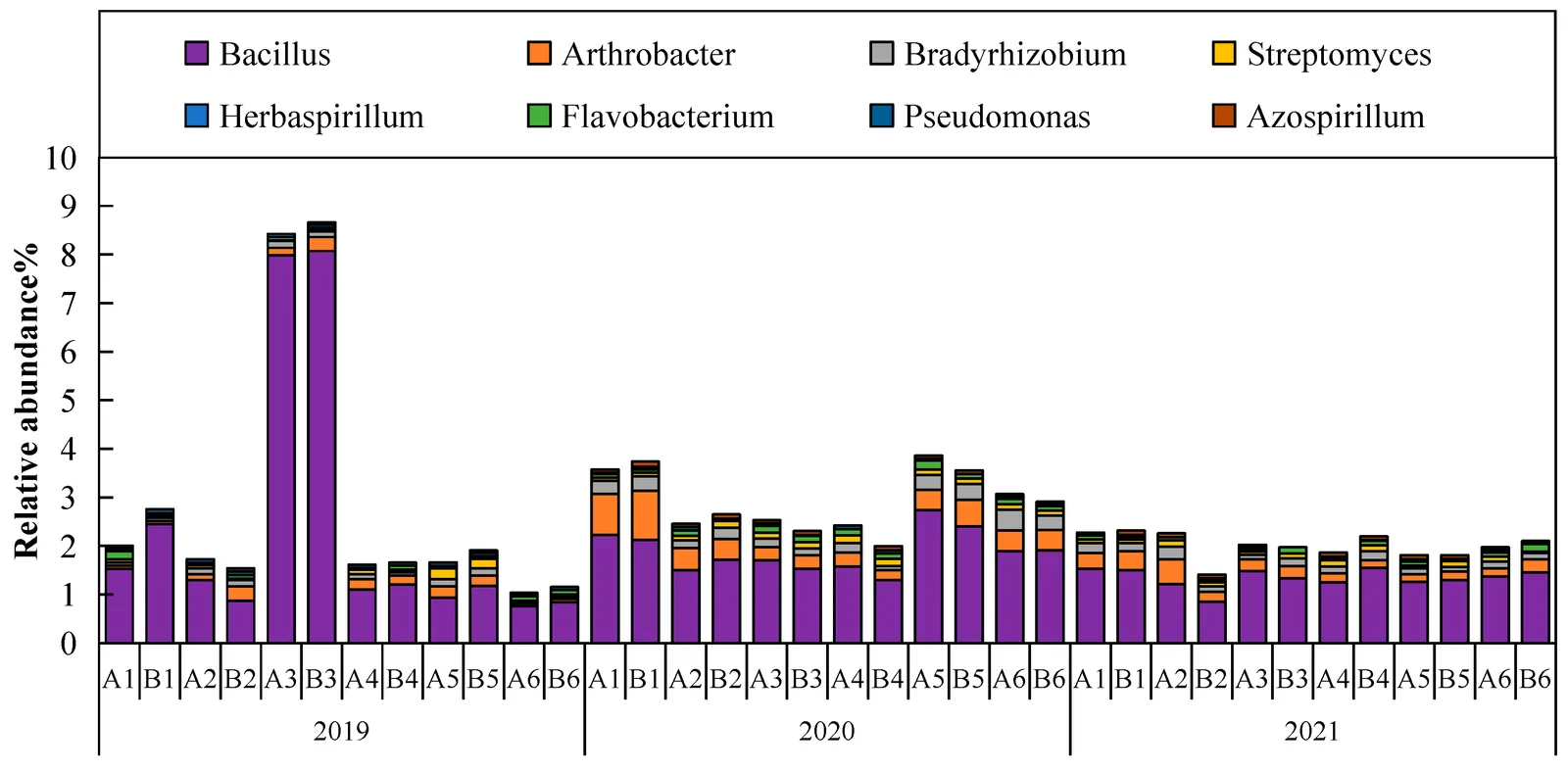
The relative abundance of rhizosphere-promoting bacteria.

On the other hand, common plant rhizosphere-promoting bacteria from the top 200 relative abundance genera were screened, mainly including Bacillus, Arthrobacter, Bradyrhizobium, Streptomyces, Herbaspirillum, Flavobacterium, Pseudomonas, and Azospirillum. The relative abundance of rhizosphere-promoting bacteria in the rhizosphere soil microorganisms of HGK60 and Zheng 58 at different growth stages over various years was compared (Figure 7). Bacillus, Rhizobium, and Rhizobium showed no significant differences between HGK60 and Zheng 58 across all growth stages from 2019 to 2021. Actinobacillus showed significant differences between HGK60 and Zheng 58 only during the seedling stage in 2019 and 2021, but in 2019, HGK60 was significantly lower than Zheng 58, while in 2021, it was the opposite. Streptomyces showed a significant advantage for HGK60 over Zheng 58 after harvest in 2019. Bacillus showed significant differences between HGK60 and Zheng 58 before sowing and after harvest in 2019, and during the heading stage in 2021, but in 2019, HGK60 was significantly higher than Zheng 58, and in 2021, it was significantly lower than Zheng 58. Pseudomonas showed a significant advantage for HGK60 over Zheng 58 only during the late growth stage in 2021. Azotobacter showed significant differences between HGK60 and Zheng 58 only during the seedling stage in 2019. Significant differences between HGK60 and Zheng 58 only occurred at certain stages, and there was no consistency or persistence. It was concluded that the planting of HGK60 had no significant effect on rhizosphere probiotics.

### 3.2. Diversity and Structure of Bacterial Communities

**Figure 8:**
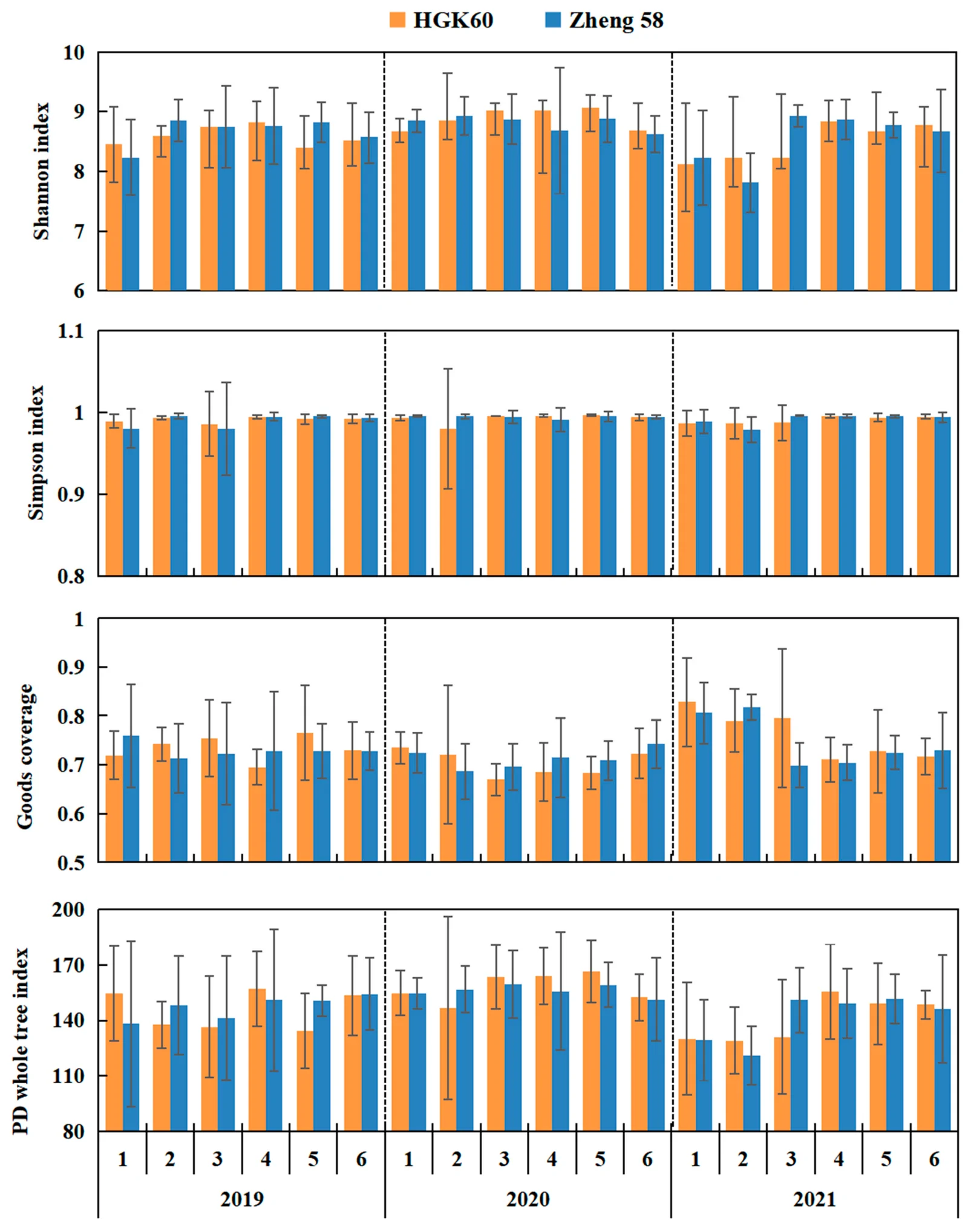
Alpha diversity analysis.

**Figure 9:**
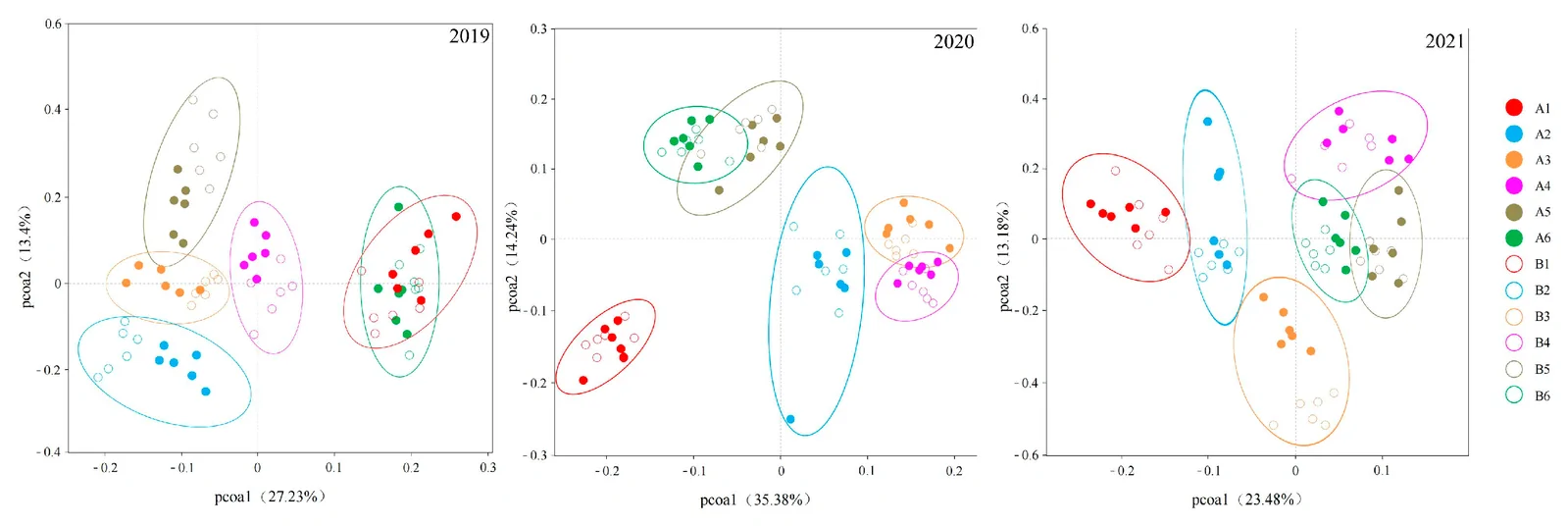
Principal coordinate analysis.

**Figure 10:**
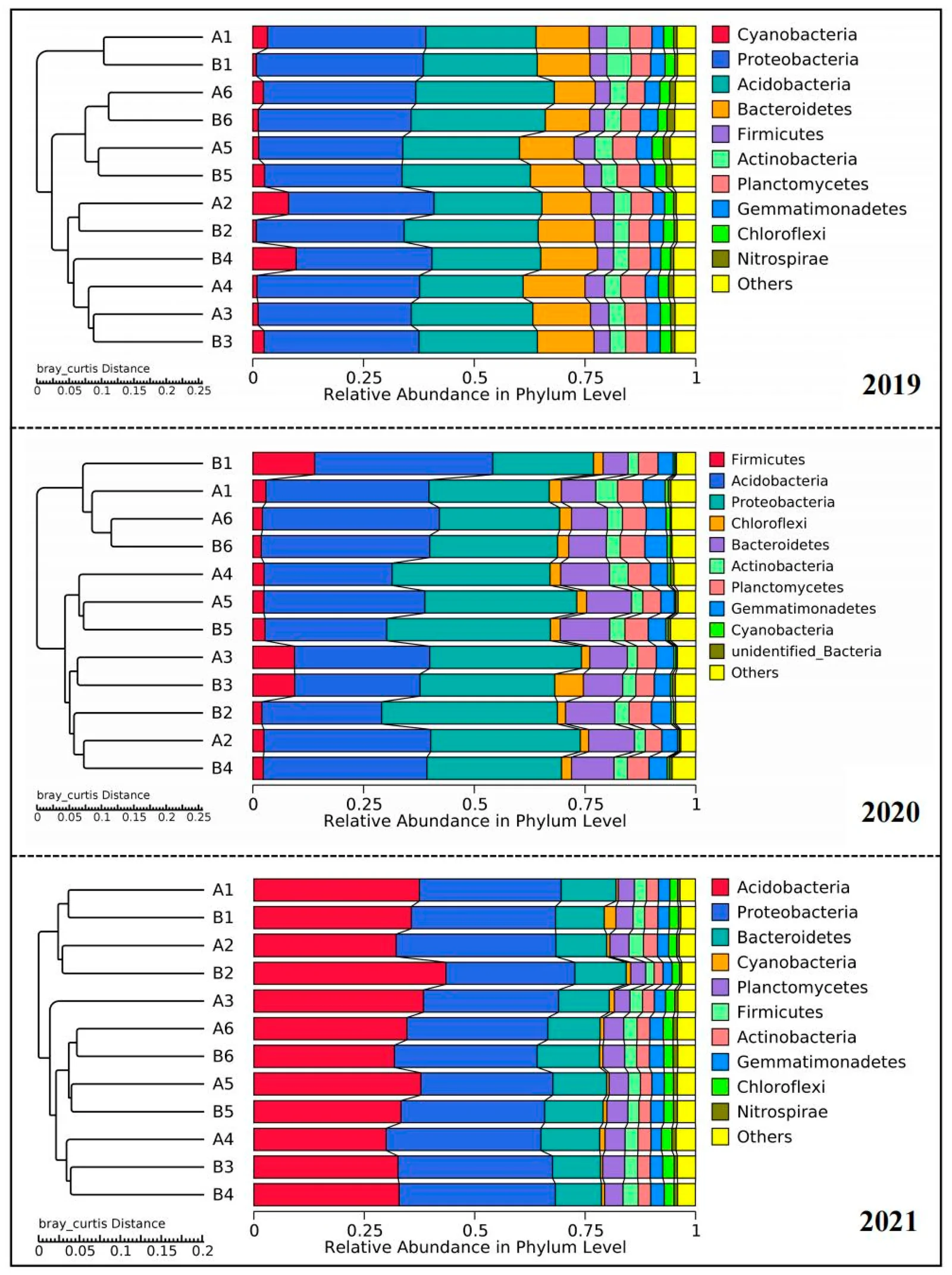
UPGMA clustering tree based on Bray–Curtis distance.

The alpha diversity index can effectively reflect the number, abundance, and distribution of species within a community. Analysis of the Shannon index, Simpson’s index, PD index, and coverage index for root soil bacterial communities at different growth stages of HGK60 and Zheng 58 (Figure 8) shows that there is no significant difference between HGK60 and Zheng 58 in any of these indices (*p* > 0.05), indicating that the planting of HGK60 has no significant impact on the alpha diversity of root soil bacteria. Beta diversity analysis is mainly used to compare differences in the overall structure of microbial communities across samples. PCoA and Anosim analysis results (Figure 9) show that in 2019, there were significant differences during the seedling stage (R = 0.383, *p* = 0.002) and the heading stage (R = 0.279, *p* = 0.011). In 2020, no significant differences were observed throughout the entire growth period. In 2021, only during the trumpet stage (R = 0.397, *p* = 0.005) did significant differences emerge. After three consecutive years of harvest, no significant differences in community structure between HGK60 and Zheng 58 were observed (*p* > 0.05). The UPGMA dendrogram based on the Bray–Curtis distance (Figure 10) shows that, except for the trumpet stage in 2021, root soil samples from all growth stages in 2019–2021 clustered together, indicating similar bacterial community structures. On the other hand, clear separation was observed across different growth stages, suggesting that growth stage is a crucial factor influencing the structure of soil bacterial communities, rather than the planting of HGK60.

## 4. Discussion

The insecticidal proteins released by genetically modified crops can enter the soil through various pathways, such as crop residues and root exudates, altering the soil environment around the crops. This, in turn, affects soil biodiversity and ultimately poses a threat to the soil ecosystem [28]. Currently, many experts have conducted risk assessments of soil ecosystems for different genetically modified crops, but the conclusions vary [4,29], and there is no clear consensus on the environmental safety issues that may arise from planting genetically modified crops.

This study employed third-generation high-throughput sequencing to investigate the impact of HGK60 planting on rhizosphere soil bacteria. Analysis of bacterial relative abundance at different taxonomic levels revealed that, overall, there were no significant differences in the phyla, classes, and orders between the rhizosphere soil bacteria of HGK60 and Zheng 58 during the same growth period. Occasional differences only appeared during certain growth periods and had a short duration, weakening to non-significant levels within a short time frame. This finding is similar to the results reported by Widmer et al. [30,31,32], who concluded that the planting of HGK60 did not have a sustained or significant effect on the relative abundance of rhizosphere soil bacteria. Additionally, this study screened for rhizosphere-promoting bacteria with relatively high abundance in corn rhizosphere soil. The analysis showed that the planting of HGK60 did not cause any significant differences in the abundance of rhizosphere-promoting bacteria such as Bacillus, Rhizobium, and Sinorhizobium. The effects on Rhizobium, Streptomyces, Pseudomonas, Pseudomonas fluorescens, and Azorhizobium were only occasional and lacked persistence and repeatability. It can be said that the planting of insect-resistant maize with the *Cry1Ah* gene in HGK60 has no significant impact on major rhizosphere-promoting bacteria. Based on current literature reviews, studies on the impact of genetically modified crop planting on rhizosphere-promoting bacteria are relatively scarce. Further research is needed to extend the study duration and broaden the scope to validate our findings.

The results of the sample complexity and comparative analysis show that the alpha diversity index of rhizosphere soil bacteria between HGK60 and Zheng 58 was not significantly different across various growth stages from 2019 to 2021. PCoA and Anosim analyses indicate that the community structure of rhizosphere soil bacteria in HGK60 and Zheng 58 during the same period was relatively similar, with little overall difference. Significant differences only briefly appeared in a certain year and growth stage, and after harvest, the differences between HGK60 and Zheng 58 were not significant. UPGMA analysis also shows that planting HGK60 did not significantly affect the beta diversity of rhizosphere soil bacterial communities [32,33]. These results all indicate that planting HGK60 does not significantly impact the diversity of corn rhizosphere soil bacteria. Although plants can alter their rhizosphere microbial communities by modifying root exudates [34,35,36], the introduction of *Cry1Ah* exogenous genes did not reach a significant level of influence on the rhizosphere soil bacteria of corn. Some existing research findings corroborate this study’s results, suggesting that genetically modified crops do not significantly affect soil microbial community diversity [37,38]. Fan et al. conducted a two-year trial on insect-resistant genetically modified corn carrying the *Cry1le* gene and found no significant impact on soil biodiversity [39]; Fazal et al.’ s study on *mcry1Ab* and *mcray2Ab* gene-transferred corn showed that its planting had minimal impact on the rhizosphere soil bacterial community, which can be disregarded [40]. In addition, the results of this study show that the growth stage is the primary factor influencing the structure of soil bacterial communities in corn rhizosphere, with significant differences observed across different growth stages, consistent with some existing research findings [32,33]. The enrichment of crop rhizosphere microorganisms is positively correlated with the degree of crop growth and development and interacts with the surrounding environment to gradually form a rhizosphere microenvironment [41]. The differences in soil rhizosphere bacterial community structures at different growth stages of corn may be due to variations in root biomass and hormone levels during different growth and developmental phases, leading to differences in root exudates [42]^.^

## 5. Conclusions

The research findings indicate that the planting of insect-resistant corn HGK60 with the *Cry1Ah* gene had no significant impact on the bacterial communities in the rhizosphere soil. The partial effects were relatively short-lived and did not persist beyond the corn harvest, and there was no reproducibility across different years. Although this study has reached relatively clear conclusions and innovatively examined the impact of insect-resistant corn HGK60 with the *Cry1Ah* gene from the perspective of rhizosphere probiotics, the environmental safety assessment of genetically modified crops is a long-term process that requires more extensive and in-depth research to ultimately scientifically determine whether GM crops pose any threat to the environment. Of course, differences between species, differences in inserted genes, variations in research methods, field management practices, and uncontrollable climatic factors can all interfere with the ecological safety studies of GM crops. It is essential to fully adhere to the “case-by-case principle” [25] and conduct follow-up studies on resistance varieties obtained through the introduction of different exogenous genes, taking into account external environmental disturbances, to facilitate the rapid development of GM breeding.

## Data Availability

The original contributions presented in this study are included in the article. Further inquiries can be directed to the corresponding authors.
